# Simultaneous intussusception associated with adenovirus infection in monozygotic twins: A case report

**DOI:** 10.1097/MD.0000000000018294

**Published:** 2019-12-20

**Authors:** Yu-Hsien Lee, Lung-Huang Lin, Shin-Pin Hung

**Affiliations:** a Department of Pediatrics, Cathay General Hospital, Taipei City; b School of Medicine, Fu Jen Catholic University, New Taipei City, Taiwan.

**Keywords:** adenovirus, intussusception, twins

## Abstract

**Rationale:**

Intussusception, a common cause of intestinal obstruction in children, typically requires medical reduction. Here, we describe the case of a pair of twins who had simultaneous intussusception and were positive for fecal adenovirus—strongly indicating that adenovirus infection may be a main cause of the intussusception.

**Patient concerns:**

Two 1-year-old twin girls were brought to Cathay General Hospital one after another on the same day. Both presented with intermittent abdominal pain, abdominal distension, diarrhea, and loss of appetite.

**Diagnoses:**

Their laboratory data were adenovirus positivity in rectal swab culture. Intussusception was diagnosed through a lower gastrointestinal series.

**Interventions:**

The twins were treated with reduction for intussusception.

**Outcomes:**

Both patients recovered well, without recurrence.

**Lessons:**

Most cases of intussusception are idiopathic. However, some potential risk factors—as strongly suggested by the current cases—are genetic factors and adenovirus infection.

## 1 Introduction

Intussusception, a common cause of acute abdomen in children aged 6 months to 2 years, is a type of intestinal obstruction occurring in pediatric emergencies. Although it sometimes resolves spontaneously, it typically requires a medical reduction. Intussusception is caused by a segment of the intestine invaginating into an adjacent segment. The classical clinical triad of intussusception includes intermittent abdominal pain, currant jelly stool, and palpable mass, but the symptoms and signs may vary in every case. Early diagnosis and appropriate therapy reduces the mortality rate to <1%. Delayed diagnosis and treatment may, however, increase its morbidity and mortality. Its symptoms include sudden onset of abdominal pain, vomiting, and bloody stool. Complications such as bowel perforation can be life-threatening. The cause of intussusception in most children is idiopathic. Nevertheless, some potential factors associated with intussusception have been mentioned in reports. Herein, we report the case of a pair of identical twins positive for fecal adenovirus and intussusception simultaneously.

## 2 Case presentation

A pair of 1-year-old identical twin girls was born prematurely at the gestational age of 35 weeks. At birth, both were healthy and had no known systemic diseases. They both received scheduled vaccinations and achieved appropriate developmental milestones.

**Figure 1:**
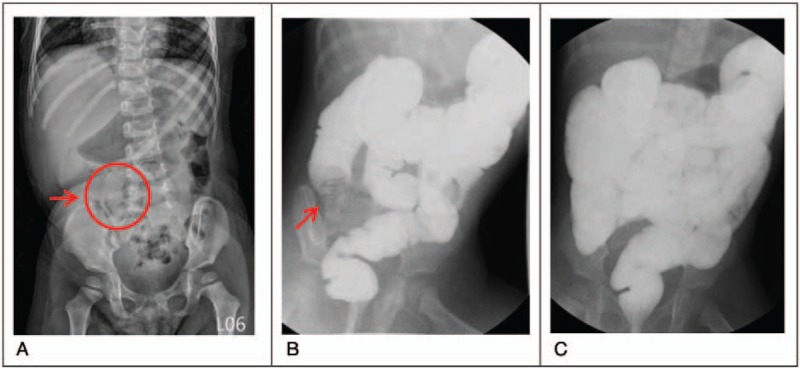
Twin A. A, Abdominal plain film revealing a mass lesion at the right upper quadrant (red arrow). B and C, Lower gastrointestinal series revealed ileocolic intussusception (red arrow) and successful reduction.

One of the twin sisters (twin A) was brought to the emergency department of Cathay General Hospital, Taiwan, with a complaint of intermittent abdominal pain at a frequency of once every 5 minutes, abdominal fullness, loose stools, and reduced appetite. Neither fever nor vomiting was mentioned. The physical examination revealed acceptable vital signs (body temperature: 37.2°C, heart rate: 100 beats/min, respiratory rate: 24 breaths/min) but a distended abdomen without muscle guarding or rebounding pain. Laboratory tests revealed mild leukocytosis with atypical lymphocytes (white blood cell count: 10.58 × 10^3^ cells/mm^3^, with 5.8% atypical lymphocytes), normal C-reactive protein level (0.111 mg/dL), presence of stool occult blood positivity (2+), absence of stool rotavirus antigen, and absence of adenovirus in throat swab. Abdominal plain radiography (Fig. 1A) revealed a mass lesion over the right upper abdomen—a positive sign for intussusception.^[1]^ Lower gastrointestinal (GI) series with water-soluble contrast medium was immediately administered; the results revealed ileocolic intussusception, with the ileal loop herniating into the proximal ascending colon (Fig. 1B). Reduction for intussusception was then performed successfully (Fig. 1C). Rectal swab culture was found positive for adenovirus 1 week later.

**Figure 2:**
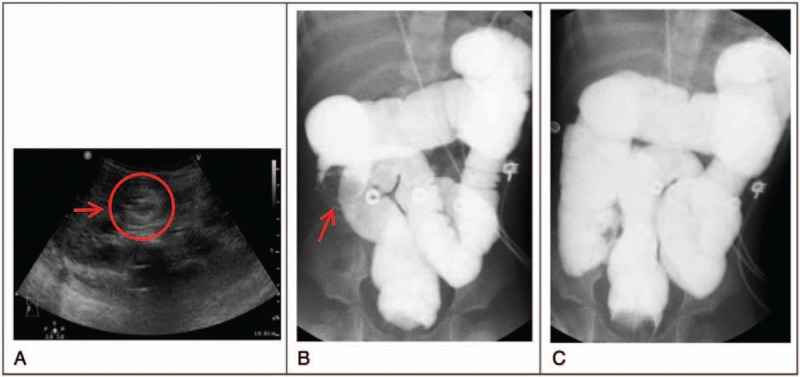
Twin B. A, Abdominal sonography displaying the target lesion (red arrow). B and C, Lower gastrointestinal series revealed ileocolic intussusception (red arrow) and successful reduction.

The sister of twin A (twin B) was brought to the emergency department approximately 8 hours after twin A was. In addition to twin A's symptoms, she had vomiting, diarrhea, and reduced activity. Laboratory tests revealed a normal white blood cell count with mildly increased atypical lymphocyte proportion (1.9%). Adenovirus throat swab provided negative results. Moreover, abdominal plain radiography revealed increased gas levels in the colon; abdominal sonography also displayed a target sign (Fig. 2A). Lower GI series exhibited ileocolic intussusception with the ileal segment herniating into the ascending and proximal transverse colon, and the reduction was performed successfully (Fig. 2B and C). Her rectal swab culture was also positive for adenovirus. Both girls recovered smoothly without relapse.

This case report was approved by our hospital institutional review board, and patient consent was obtained.

## 3 Discussion

Intussusception is a common cause of intestinal obstruction in children younger than 3 years. Although the annual incidence of intussusception varies by country and year, a retrospective study reported that the incidence rate of intussusception in Taiwan was approximately 0.77 per 1000 live births, with the highest incidence noted between 12 and 24 months of age.^[2]^ According to the *Brighton* Collaboration *criteria*, detailed history taking, physical examination, abdominal ultrasound, and radiological examination (eg, abdominal plain film, air or liquid contrast enema, and computed tomography) are required for diagnosis.^[3]^ Early diagnosis and appropriate therapy are essential to preventing morbidity and mortality. Most intussusception cases in children remain idiopathic. In clinical settings, earlier recognition of its causes and risk factors results in superior treatment and disease prevention. Therefore, many physicians have published studies identifying suspicious causes of intussusception and analyzing intussusception cases in children. Our hospital published a study on the association between intussusception and adenovirus infection in children, where we analyzed the annual rates of intussusception and adenovirus infection from January 2008 to June 2011 and revealed similar peak incidence months for both diseases.^[4]^ Several studies have also noted the potential factors associated with intussusception such as the presence of a lead point, infection, familial tendency, and food allergy.^[5–10]^ Of these, adenovirus was the most predominant infectious pathogen detected in intussusception.^[11–13]^ Guo et al^[14]^ also collected 191 cases of 1 or multiple intussusception recurrences and reported some potential risk factors, such as age, duration of symptoms, mass location, and a pathological lead point, for recurrent intussusception in children. A study of 12 Mexican children who underwent surgical resection for intussusception revealed that the adenovirus antigen in epithelial cells was detected in 4 (33%) of the tissue specimens through immunohistochemical analysis. Furthermore, the species C (nonenteric) adenovirus was the most predominant type.^[15]^ Okimoto et al^[13]^ analyzed 71 stool samples with adenovirus and discovered that nonenteric adenovirus are particularly more likely to be associated with intussusception. Jang et al^[12]^, however, analyzed the association between adenovirus subgroups and fecal or respiratory adenoviral epidemic trends and reported that intussusception is associated with GI involved, but not respiratory-involved, nonenteric adenovirus. This may explain why both of our patients presented GI symptoms without respiratory symptoms, with both of their throat swabs negative but rectal swabs positive for adenovirus. Adenovirus subgroup analysis was, however, not performed in our twins. Several reports have discussed intussusception occurring in twins or family members.^[7–9,16,17]^ Simultaneous intussusception in twins or siblings, however, implies that genetic predisposition may not be the only factor responsible for intussusception.^[9,16,17]^ In our report, a pair of identical twins was diagnosed as having intussusception, and their stool culture both were positive for adenovirus. This suggested that the intussusception was associated with genetic causes and likely triggered by infection because of the short duration between these cases. Moreover, laboratory data supported our hypothesis that adenovirus is the most likely causative agent pathogen in our cases.

Adenovirus infection in infancy often results in silent infection but may be associated with lymphoid hyperplasia, including the lymphoid tissue in the intestine. Very rarely such lymphoid hyperplasia may act as a focus for intussusception. In Taiwan, Clarke revealed that the proportion of adenovirus infection in patients with intussusception was approximately 10 times that in controls. Twenty-six percent of the patients showed adenovirus infection as evidenced by recovery from throat swab, rectal swab, or mesenteric node removed at surgery.^[18]^ This strongly suggested that adenovirus may be an etiologic agent associated with intussusception. These cases involved identical twins; thus, the confounding due to genetic differences can be ruled out. Intussusception in our twins occurred simultaneously, and both of their feces were confirmed to contain adenovirus, which may also be strong evidence that adenovirus is a leading cause of intussusception.

## 4 Conclusion

Although most intussusception cases are idiopathic, factors such as a lead point near the ileocecal junction, bacterial and viral infections, and familial tendencies may be potential causes. We reported a set of twins who both presented intussusception within 8 hours of each other, and this timetable was similar to that in reports of intussusception between identical twins, which was typically within 48 hours of each other.^[9]^ In our cases also, we verified adenovirus positivity through rectal swab culture, which was compatible with studies that considered adenovirus infection to be a potential trigger of intussusception.^[4,11–13,15]^ Because the illness occurred within a relatively short time interval between the twins, our case results strongly suggests that intussusception has a genetic cause and that it can be triggered through adenovirus infection.

## Author contributions

**Supervision:** Lung-Huang Lin, Shin-Pin Hung.

**Writing – original draft:** Yu-Hsien Lee.

**Writing – review and editing:** Yu-Hsien Lee, Lung-Huang Lin.
